# Serous cystadenocarcinoma of the pancreas: a population-based study

**DOI:** 10.1186/s12957-026-04604-z

**Published:** 2026-09-28

**Authors:** Theodore Dotevall, Emelie Kolmodin, Ebba Thylefors, Roland Andersson, Daniel Ansari

**Affiliations:** Department of Surgery, Clinical Sciences Lund, Lund University, Skåne University Hospital, Lund, Sweden

**Keywords:** Pancreatic cancer, Pancreatic cystic neoplasms, Serous cystadenocarcinoma, Serous cystic neoplasms, SEER

## Abstract

**Background:**

Serous cystadenocarcinoma (SCAc) of the pancreas is an exceptionally rare malignant pancreatic neoplasm, and its clinical characteristics, treatment patterns, and prognosis remain poorly defined. The aim of this population-based study was to characterize the demographic and clinical features, treatment patterns, and survival of patients with pancreatic SCAc.

**Methods:**

Patients with microscopically confirmed SCAc were identified from the Surveillance, Epidemiology, and End Results (SEER) registry. Demographic, tumor-related, treatment, and survival data were analyzed using descriptive statistics and Kaplan-Meier survival analysis.

**Results:**

Twenty-one patients with SCAc were identified. Median age at diagnosis was 69 years, and 13 patients (62%) were female. Tumors most frequently involved the pancreatic body or tail. Disease was localized in 4 patients (19%), regional in 6 (29%), distant in 9 (43%), and unstaged in 2 (10%). Surgical resection was performed in 12 patients (57%), and 6 (29%) received chemotherapy. Estimated 5-year overall survival (OS) was 60.5% for the overall cohort. Five-year OS was 73.3% among patients who underwent resection and 44.4% among those who did not.

**Conclusions:**

Pancreatic SCAc is a rare malignancy with frequent involvement of the pancreatic body or tail. Surgical resection was the most common treatment, whereas chemotherapy was used in a minority of patients. Population-based data suggest relatively favorable long-term survival.

## Introduction

Serous cystic neoplasms of the pancreas are uncommon epithelial neoplasms that are predominantly benign. They include serous cystadenoma and the exceptionally rare malignant counterpart, serous cystadenocarcinoma (SCAc) [1, 2]. While serous cystadenoma is generally indolent, SCAc can exhibit locally invasive and metastatic behavior, but its clinical course and biological characteristics remain poorly understood.

SCAc was first described by George et al. in 1989, who reported a serous pancreatic neoplasm with both local invasion and distant metastases [3]. Subsequent reports have confirmed the existence of malignant serous neoplasms but have also highlighted substantial difficulties in distinguishing SCAc from serous cystadenoma. The primary tumor may be morphologically indistinguishable from a benign serous neoplasm, and malignant behavior may only become evident through subsequent invasion or metastasis [4, 5].

The pathological classification of SCAc has therefore remained challenging. Reid et al. demonstrated considerable heterogeneity among previously reported cases of “serous cystadenocarcinoma” and emphasized the importance of unequivocal evidence of malignant behavior when classifying a serous neoplasm as malignant [5]. The World Health Organization (WHO) classification recognizes serous neoplasms of the pancreas and defines malignancy by malignant behavior, with distant metastasis providing the clearest criterion for SCAc [2]. The distinction is particularly relevant for tumors showing local invasion without distant metastases, for which the biological significance remains uncertain [5, 6].

The natural history of SCAc is also incompletely characterized. Case reports have described delayed development of metastatic disease years after an initial diagnosis of serous cystadenoma, suggesting that malignant behavior may emerge after a prolonged period of apparently indolent disease [7]. However, the available evidence has largely been derived from individual case reports and small case series, limiting the ability to define the typical presentation, treatment, and prognosis of SCAc.

A recent systematic review by Wilhelm et al. represents the most comprehensive synthesis of the published SCAc literature to date [8]. However, the available evidence remains based exclusively on published cases and is therefore susceptible to selection and publication bias. A further systematic review by Tan et al. has highlighted the continuing uncertainty surrounding locally invasive serous neoplasms and their potential for subsequent metastatic disease [6]. Thus, population-based data on SCAc remain lacking.

The Surveillance, Epidemiology, and End Results (SEER) Program provides an opportunity to examine this exceptionally rare malignancy in a population-based setting. The aim of this study was to provide the first population-based characterization of pancreatic SCAc, including its demographic and clinicopathological characteristics, stage at diagnosis, treatment patterns, and overall survival.

## Methods

### Data source and study population

Data were obtained from the Surveillance, Epidemiology, and End Results (SEER) Program of the National Cancer Institute [9]. The SEER database collects population-based cancer incidence and survival data from geographically defined cancer registries in the United States and includes information on patient demographics, primary tumor characteristics, disease stage, treatment, and survival outcomes. The SEER 2004–2023 dataset was used for the present study.

Patients with pancreatic SCAc were identified using the International Classification of Diseases for Oncology, Third Edition (ICD-O-3). Cases were eligible if they fulfilled both of the following criteria: (1) histological diagnosis of serous cystadenocarcinoma (ICD-O-3 histology code 8441/3), and (2) primary tumor site within the pancreas (ICD-O-3 topography codes C25.0-C25.9). Only cases with microscopic confirmation of the diagnosis were included. Cases without microscopic confirmation were excluded to increase diagnostic specificity and minimize the inclusion of clinically or radiologically suspected cases.

The following variables were extracted from the SEER database: age at diagnosis, sex, primary tumor location, SEER summary stage, treatment with surgery and chemotherapy, survival time, and vital status. Tumor location was categorized according to the recorded primary site as pancreatic head, body, tail, overlapping lesion, or pancreas not otherwise specified. SEER stage was categorized as localized, regional, distant, or unstaged according to the classification available in the dataset.

### Statistical analysis

Statistical analyses were performed using Stata/MP 18.0. Categorical variables, including sex, primary tumor location, SEER stage, surgical treatment, and chemotherapy, were summarized as frequencies and percentages. Age at diagnosis was summarized as the median with interquartile range (IQR). Overall survival (OS) was defined as the time from diagnosis to death from any cause, with patients alive at last follow-up censored at that time. Kaplan-Meier analysis was used to estimate OS and the 5-year survival probability. Given the rarity of SCAc and the small number of identified cases, analyses were primarily descriptive, and no univariable or multivariable survival analyses were performed.

## Results

### Patient and tumor characteristics

**Fig. 1:**
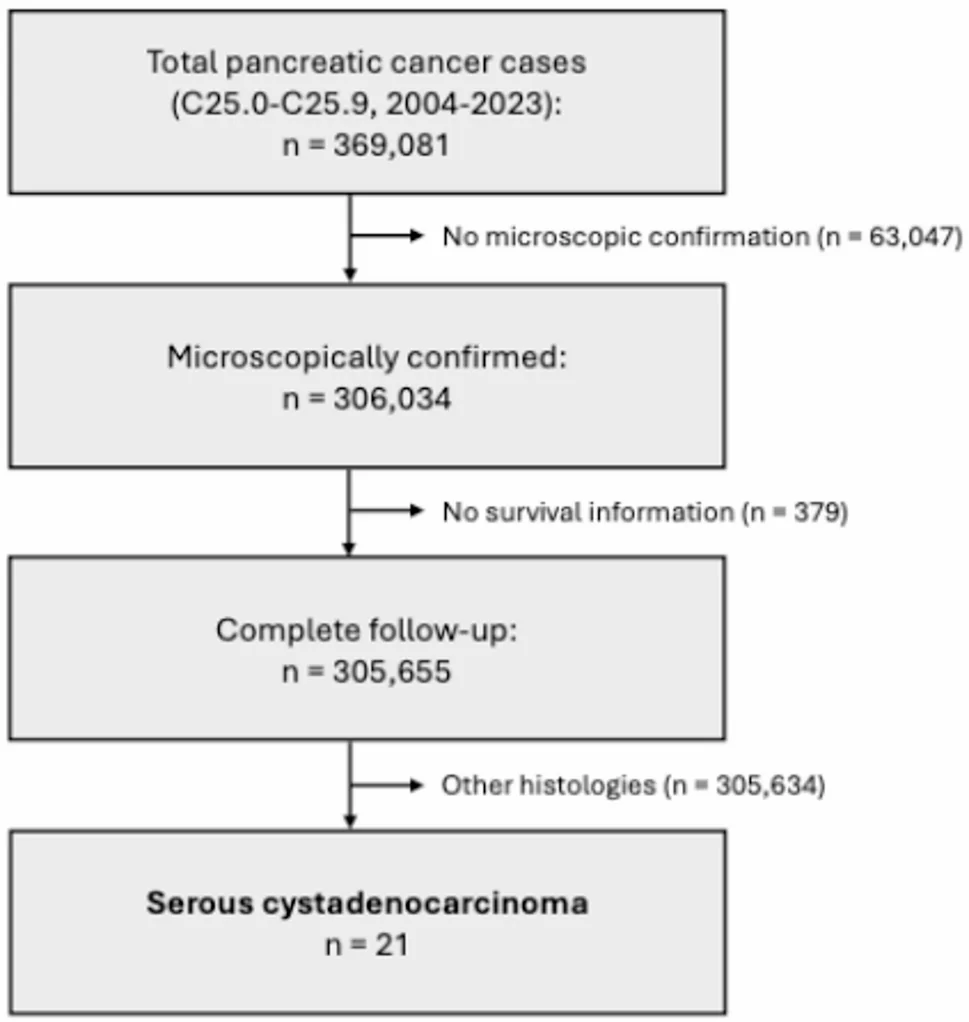
Flowchart of patient selection from the SEER database

A total of 21 patients with microscopically confirmed pancreatic SCAc were identified in the SEER registry after application of the predefined inclusion and exclusion criteria (Fig. 1). The median age at diagnosis was 69 years, and 13 patients (62%) were female.

Tumors were distributed across several pancreatic regions. The most common tumor location was the pancreatic body or tail, accounting for 8 cases (38%). Five tumors (24%) were located in the pancreatic head, 3 (14%) were classified as overlapping lesions, and the primary tumor location was unspecified in 5 patients (24%).

According to SEER stage, 4 patients (19%) had localized disease, 6 (29%) had regional disease, and 9 (43%) had distant disease at diagnosis. Two patients (10%) had an unstaged tumor. Thus, distant disease was present in the largest proportion of patients at diagnosis.

### Treatment

Surgical resection was performed in 12 patients (57%), while 6 patients (29%) received chemotherapy. Patient demographics, tumor characteristics, SEER stage, and treatment are summarized in Table 1.

**Table 1:** Demographic, clinical, and treatment characteristics of patients with pancreatic serous cystadenocarcinoma in the SEER cohort

Characteristic	*n* (%) or median (IQR)
Age, median (IQR)	69 (55–75)
Female sex	13 (62%)
Tumor location	
Head	5 (24%)
Body/tail	8 (38%)
Overlapping lesion	3 (14%)
Not specified	5 (24%)
SEER stage	
Localized	4 (19%)
Regional	6 (29%)
Distant	9 (43%)
Unknown	2 (9%)
Treatment	
Surgical resection	12 (57%)
Chemotherapy	6 (29%)

Data are presented as *n* (%) unless otherwise indicated

*Abbreviations*: *IQR* Interquartile range, *SEER* Surveillance, Epidemiology, and End Results

### Overall survival

**Fig. 2:**
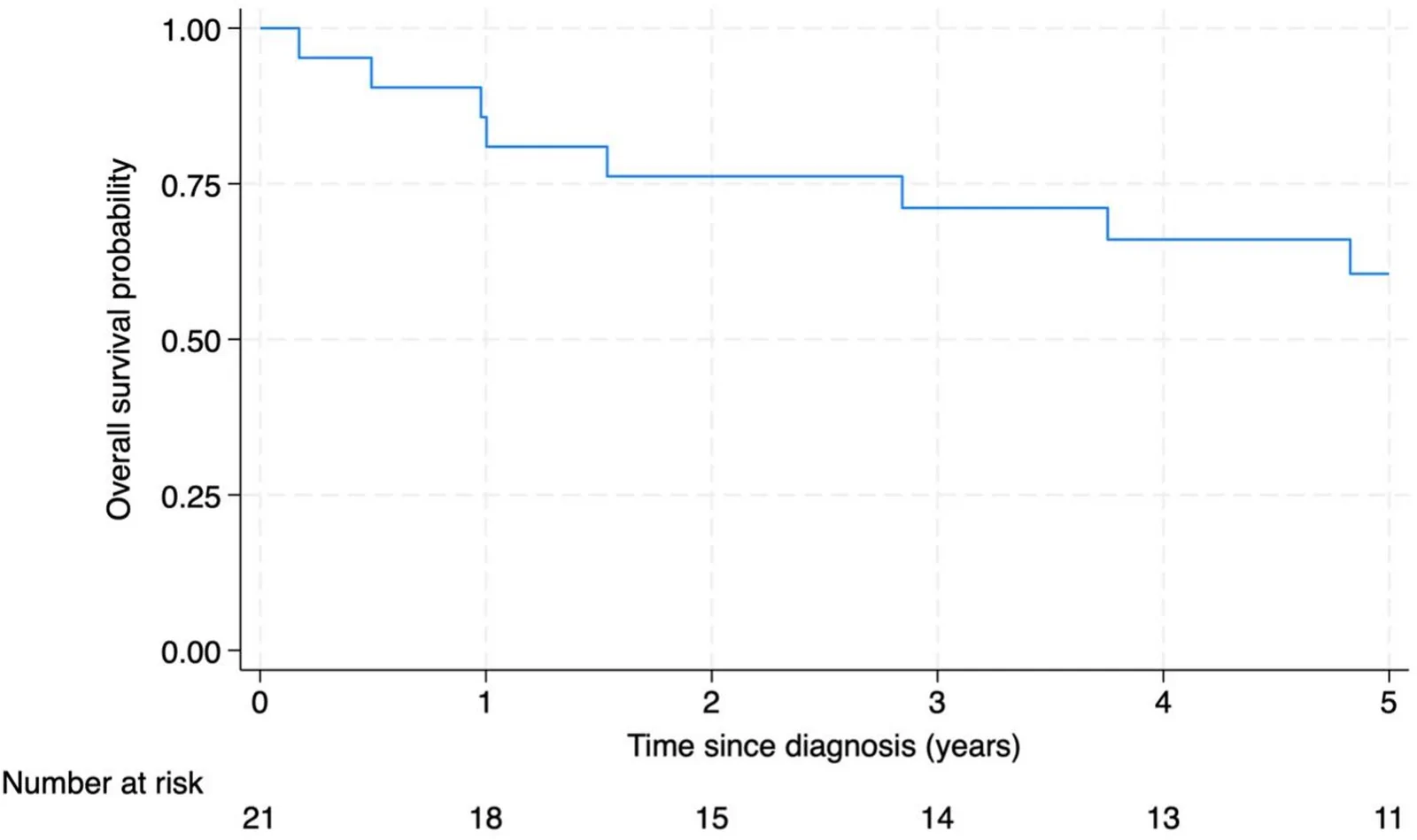
Overall survival of patients with pancreatic serous cystadenocarcinoma in the SEER cohort

The estimated 5-year overall survival (OS) for the cohort was 60.5% (Fig. 2). The estimated 5-year OS was 73.3% among patients who underwent surgical resection and 44.4% among those who did not undergo resection.

## Discussion

In this first population-based study of pancreatic SCAc, 21 patients with microscopically confirmed disease were identified in the SEER registry. The cohort demonstrated a slight female predominance, a median age at diagnosis of 69 years, and a predominance of tumors located in the pancreatic body or tail. At diagnosis, 43% of patients had distant disease, while 57% underwent surgical resection and 29% received chemotherapy. The estimated 5-year overall survival was 60.5%. Given the extreme rarity of SCAc, these population-based data complement the predominantly case-based literature and provide a broader perspective on the presentation and outcomes of this unusual pancreatic malignancy.

The demographic characteristics of the SEER cohort are broadly consistent with previous reports. In the largest multinational study of pancreatic serous cystic neoplasms, Jais et al. evaluated 2,622 patients and reported a marked female predominance and a median age of 58 years, although only three patients had serous cystadenocarcinoma [1]. Earlier SCAc-specific literature has similarly suggested a predominance among women and presentation in the seventh decade. King et al. identified 25 cases in a structured review of the literature, with a mean age of 68 years and 60% of patients being female [10]. In a subsequent systematic review, Huh et al. identified 27 malignant serous cystic neoplasms, reporting a mean age of 68.0 years, with 74% of patients being female [4]. More recently, Wilhelm et al. identified 38 histologically confirmed SCAc cases from 36 publications in a systematic review, reporting a median age of 66.5 years and 71% of patients being female [8]. The present cohort, with a median age of 69 years and 62% female patients, is therefore broadly concordant with the accumulated literature.

Tumor location was also comparable with previous reports. In the present cohort, the pancreatic body or tail was the most common location, accounting for 38% of cases, followed by the pancreatic head in 24%. Wilhelm et al. similarly reported a predominance of tumors in the body or tail [8]. This distribution is consistent with the broader pattern observed for serous cystic neoplasms [1, 11]. However, the relatively large proportion of tumors with an unspecified primary site in SEER highlights an important limitation of registry data and warrants caution when interpreting anatomical distribution.

The extent of disease at diagnosis provides an interesting contrast between the present population-based cohort and the published case literature. Distant disease was present in 43% of patients in the SEER cohort compared with 68% (26/38) in the systematic review by Wilhelm et al. [8]. Despite the high prevalence of metastatic disease, all patients in the Wilhelm series underwent surgical resection, illustrating the distinctive treatment approach reported for this rare tumor. This finding should not be interpreted as evidence that distant disease is generally not a contraindication to surgery, but rather reflects the highly selected nature of the published SCAc literature and the reported willingness to pursue resection in selected patients, including some with metastatic disease. The lower proportion of distant disease in the SEER cohort may therefore reflect differences in case ascertainment and selection between population-based and published case series. However, differences in staging, diagnostic criteria, and reporting also limit direct comparison between the two cohorts.

The distinction between benign serous cystic neoplasms and SCAc remains a central diagnostic challenge. SCAc was originally described by George et al. in 1989 in a tumor that was histologically similar to serous cystadenoma but demonstrated malignant behavior through local invasion and metastases [3]. Subsequent reports have emphasized that the primary tumor may be morphologically indistinguishable from a benign serous cystadenoma, with malignant behavior providing the principal evidence of malignancy [4, 5, 10]. This distinction is particularly important for registry-based studies because centralized pathological review is not possible and classification depends on the diagnosis recorded in the underlying clinical and pathology records.

The definition of SCAc has also evolved. Huh et al. characterized malignant serous cystic neoplasms by local invasion and/or distant metastasis [4], whereas Reid et al. subsequently re-evaluated the pathology of 193 serous neoplasms and questioned the classification of some historical cases of SCAc, particularly those diagnosed solely on the basis of local adhesion or invasion without convincing evidence of metastatic disease [5]. The current World Health Organization classification recognizes serous cystic neoplasms as a distinct pancreatic epithelial neoplasm, with malignant behavior being central to the diagnosis of carcinoma [2]. These evolving criteria create an important source of potential heterogeneity in both historical case reports and registry-based datasets.

This issue has been further highlighted by the recent study of Tan et al., who systematically evaluated locally invasive pancreatic serous cystic neoplasms [6]. Among 25 patients, infiltrative growth was present in 23, local invasion occurred in 40%, and 28% subsequently developed distant metastases despite having no metastases at initial presentation. These findings suggest that locally invasive serous neoplasms may represent a biologically aggressive subgroup and that malignant potential may become evident only during longitudinal follow-up. Consequently, registry-based studies using an ICD-O-3 diagnosis of SCAc should be interpreted in the context of evolving pathological definitions and the possibility of historical diagnostic heterogeneity.

Treatment patterns in the present study support the central role of surgery in the management of SCAc. More than half of the SEER patients underwent resection, whereas chemotherapy was recorded in fewer than one-third. This is broadly consistent with the published literature. All patients in the systematic review by Wilhelm et al. underwent surgical resection, while chemotherapy was rarely used [8]. Earlier reviews likewise identified surgery as the principal treatment approach [4, 10]. Individual reports have described prolonged survival after aggressive surgical treatment, including in patients with metastatic disease [12–15]. However, there is no established systemic therapy specifically for SCAc, and available evidence remains limited to isolated cases and small series. Treatment is therefore generally individualized according to tumor extent, resectability, patient characteristics, and clinical circumstances.

The estimated 5-year overall survival in the present SEER cohort was 60.5%. Although this estimate is inherently imprecise because of the small sample size, it is broadly consistent with published reports suggesting that SCAc may have a relatively indolent course despite its capacity for local invasion and distant metastasis. Wilhelm et al. similarly reported prolonged survival among patients with SCAc, particularly following resection [8], while earlier case reports have described long-term survival even in selected patients with metastatic disease [10, 13, 16]. Direct comparison with the case-based literature is nevertheless limited by selection bias, variable follow-up, and inconsistent reporting of survival outcomes. The combination of metastatic potential and relatively favorable survival may reflect the underlying biology of serous neoplasms, which are generally indolent lesions with low disease-related mortality [1, 11]. The morphological similarity between benign and malignant serous neoplasms, together with the observation that some locally invasive tumors develop distant metastases only after prolonged follow-up, further suggests a biological continuum between indolent and malignant disease [5, 6]. These findings support continued surveillance after resection, although the optimal duration and intensity remain undefined.

Several limitations should be considered when interpreting these findings. The small number of patients reflects the extreme rarity of SCAc and limits the precision of survival estimates and the assessment of prognostic factors or treatment effects. In addition, SEER lacks detailed clinicopathological, molecular, and treatment information, and centralized pathological review was not possible, which is particularly relevant given the evolving definition of SCAc [2, 5, 6]. Nevertheless, the population-based design is an important strength, providing a standardized assessment of demographic characteristics, tumor distribution, treatment patterns, and survival while reducing some of the selection inherent in case reports and institutional series. Thus, the present study complements the predominantly case-based literature and provides a broader population-level perspective on the clinical characteristics and outcomes of this exceptionally rare malignancy.

In conclusion, pancreatic SCAc is an exceptionally rare malignancy with demographic and anatomical characteristics broadly consistent with previous case-based reports. Although distant disease was present in a substantial proportion of patients, long-term survival was observed in this population-based cohort, supporting the impression that SCAc may exhibit a more indolent course than many other pancreatic malignancies. Surgical resection remains the predominant treatment approach, whereas the role of systemic therapy remains uncertain. The major challenges in studying SCAc are its extreme rarity and the evolving distinction between benign, locally invasive, and unequivocally malignant serous neoplasms. International collaboration and standardized collection of clinicopathological, molecular, treatment, and long-term outcome data will be important to better define its biology and establish appropriate treatment and surveillance strategies.

## Data Availability

The datasets analyzed during the current study are available from the corresponding author on reasonable request.
